# Supplementary material for: Iron Supplementation Delays Aging and Extends Cellular Lifespan through Potentiation of Mitochondrial Function
Source: Cells. 2022 Mar 2;11(5):862. doi: 10.3390/cells11050862 (PMC8909192; doi:10.3390/cells11050862)
Supplement: Supplementary file 1 [file cells-11-00862-s001.zip › cells-1566375-supplementary.pdf]

# Supplementary material

## Iron supplementation delays aging and extends cellular lifespan through potentiation of mitochondrial function

Jovian Lin Jing 1,†, Trishia Cheng Yi Ning 1,†, Federica Natali 2,3,

Frank Eisenhaber 1,3,4,* and Mohammad Alfatah 1,*

1 Bioinformatics Institute (BII), A*STAR, Singapore 138671, Singapore;
linjj@intern.bii.a-star.edu.sg (J.L.J.);
chengynt@intern.bii.a-star.edu.sg (T.C.Y.N.)

2 Singapore Institute of Food and Biotechnology Innovation (SIFBI),
A*STAR, Singapore 138669, Singapore;
natali_federica_from.tp@sifbi.a-star.edu.sg

3 School of Biological Sciences (SBS), Nanyang Technological University
(NTU), Singapore 637551, Singapore

4 Genome Institute of Singapore (GIS), A*STAR, Singapore 138672, Singapore

* Correspondence: alfatahm@bii.a-star.edu.sg (M.A.);
franke@bii.a-star.edu.sg (F.E.)

† These authors contributed equally to this work.

**Keywords**

Iron; Chronological aging; Cellular lifespan extension; Mitochondria; AMPK;
*Saccharomyces cerevisiae*

**Table S1.** List of primers used for RT-PCR in this study.

| Primer | Sequence (5' -> 3') | Gene | Primer direction |
|---|---|---|---|
| MA71 | GAAGTGTGATGTCGATGTCC | ACT1 | Forward |
| MA72 | TCTTTCTGGAGGAGCAATG | ACT1 | Reverse |
| MA89 | TTCTGGTTTAGAAATGGCAC | GDH1 | Forward |
| MA90 | TTGACCAAAGATGGCAAG | GDH1 | Reverse |
| MA101 | GTTGGTCTCCACCATTTATG | CIT1 | Forward |
| MA102 | TGGCAACACCAAACAATAC | CIT1 | Reverse |
| MA144 b | TGGGTGATTTGAACAAAAAG | PYC1 | Forward |
| MA145 | GTGCACCAATGTGTAATGG | PYC1 | Reverse |
| MA146 | ATGGCTCAGCGTAAGTTTG | CIT2 | Forward |
| MA147 | GACACCAGAGTGAGCATCC | CIT2 | Reverse |
| MA148 | TGCTTTGGAACCAAGATTC | ACO1 | Forward |
| MA149 | TATCGATTCTGTCATCAGGG | ACO1 | Reverse |
| MA152 | GATGGAACGGAAGTGCTAC | SDH2 | Forward |
| MA153 | AACGGTAGGCTTGCATTAG | SDH2 | Reverse |
| MA202 | GACAGGAGATGTTTGACCG | AAT1 | Forward |
| MA203 | CGACAATCTACCATCACCTG | AAT1 | Reverse |
| MA204 | GGGATGTTCTCCTTTACAGG | AAT2 | Forward |
| MA205 | GCACCACTTCATCAATGG | AAT2 | Reverse |
| MA206 | TTTGCCAACGAATCTGAC | ACO2 | Forward |
| MA207 | TTTGCCTTGATGGTGAAC | ACO2 | Reverse |
| MA210 | TCATGATCGCCAATCTATTG | FUM1 | Forward |
| MA211 | GGTTCAAAGCGGTGACTAAC | FUM1 | Reverse |
| MA212 | TCAAACCAATTGTCCTTCAC | GDH2 | Forward |
| MA213 | TTTCCAATACGGACTGAGG | GDH2 | Reverse |
| MA214 | TATCCGGTCTGGAAATGG | GDH3 | Forward |
| MA215 | AATGATGGCAAGGTGTTTG | GDH3 | Reverse |
| MA220 | CGGTACCATCTTAGGCAAC | IDH1 | Forward |
| MA221 | TCATGGCAGTTGGGTTAG | IDH1 | Reverse |
| MA222 | TATTGCCGGTCAAGATAAAG | IDH2 | Forward |
| MA223 | GCCAAGTCACCTGTTCTG | IDH2 | Reverse |
| MA224 | GTACGAAAGAGGAAACCAAGAG | KGD1 | Forward |
| MA225 | TTAATGAATCACGTAGCTGAGC | KGD1 | Reverse |

| | | | |
|---|---|---|---|
| MA226 | TCAATTCACCACAAACAGC | KGD2 | Forward |
| MA227 | CAGTCTTCAAGAAGGTAACAGC | KGD2 | Reverse |
| MA228 | CGAAGCTGCTCAATTTCTC | LSC1 | Forward |
| MA229 | TTTGGATTCTGCATCAGTTC | LSC1 | Reverse |
| MA230 | ATTCCTGGAGGGACTTATCAC | LSC2 | Forward |
| MA231 | GATCGCCTCCATTTAATTTG | LSC2 | Reverse |
| MA232 | ACCATCACCAACACCTCTG | MAE1 | Forward |
| MA233 | TGACCCACTGTAAACATTCG | MAE1 | Reverse |
| MA234 | TCGAATTCTTTGCATCTCC | MDH1 | Forward |
| MA235 | AGCAACAAAGTTGACACCC | MDH1 | Reverse |
| MA236 | TGATGTCCACGATACTCACC | PYC2 | Forward |
| MA237 | ATCTGCTGGTGAAGAGACAAC | PYC2 | Reverse |
| MA238 | TGATGATGTGAAGACGACC | SDH1 | Forward |
| MA239 | AATCCTCTCTTGCATGAGC | SDH1 | Reverse |
| MA242 | CCTACAACCAGGATACCGTC | SOD2 | Forward |
| MA243 | CATCGAATCTTCTGGATGC | SOD2 | Reverse |
| MA296 | TGCTATCGGCGATTGTAC | NDE1 | Forward |
| MA297 | CTTCTGAATCGTCTTTGGC | NDE1 | Reverse |
| MA298 | GATTTAGGTGCCTTAGCATACC | NDI1 | Forward |
| MA299 | CCTTTAATCTCGATCTTGCAG | NDI1 | Reverse |
| MA300 | AACCCTTACTTCCCAGGTG | CYT1 | Forward |
| MA301 | TTTCTTTCGTCATGTTCAGG | CYT1 | Reverse |
| MA302 | GAGTCAAAGACCCTCAATGG | RIP1 | Forward |
| MA303 | GCAGGTCCCTTTCTGATTC | RIP1 | Reverse |
| MA304 | TGATTTAGTTCCTGCTCCTG | COX6 | Forward |
| MA305 | CATCCTTCAATTCATCCAAG | COX6 | Reverse |
| MA306 | AAATCTTCCAATCTTCCAC | COX7 | Forward |
| MA307 | CCTTCTTGGCTTTGATAC | COX7 | Reverse |
| MA308 | GCCTCCAAACAATTACAAGTG | ATP7 | Forward |
| MA309 | GTCGTCAACAGTTAATTCATCG | ATP7 | Reverse |
| MA310 | TCAAGGCTAACACTAGATTGG | ATP17 | Forward |
| MA311 | CTCTTCCGCACCTTTATG | ATP17 | Reverse |

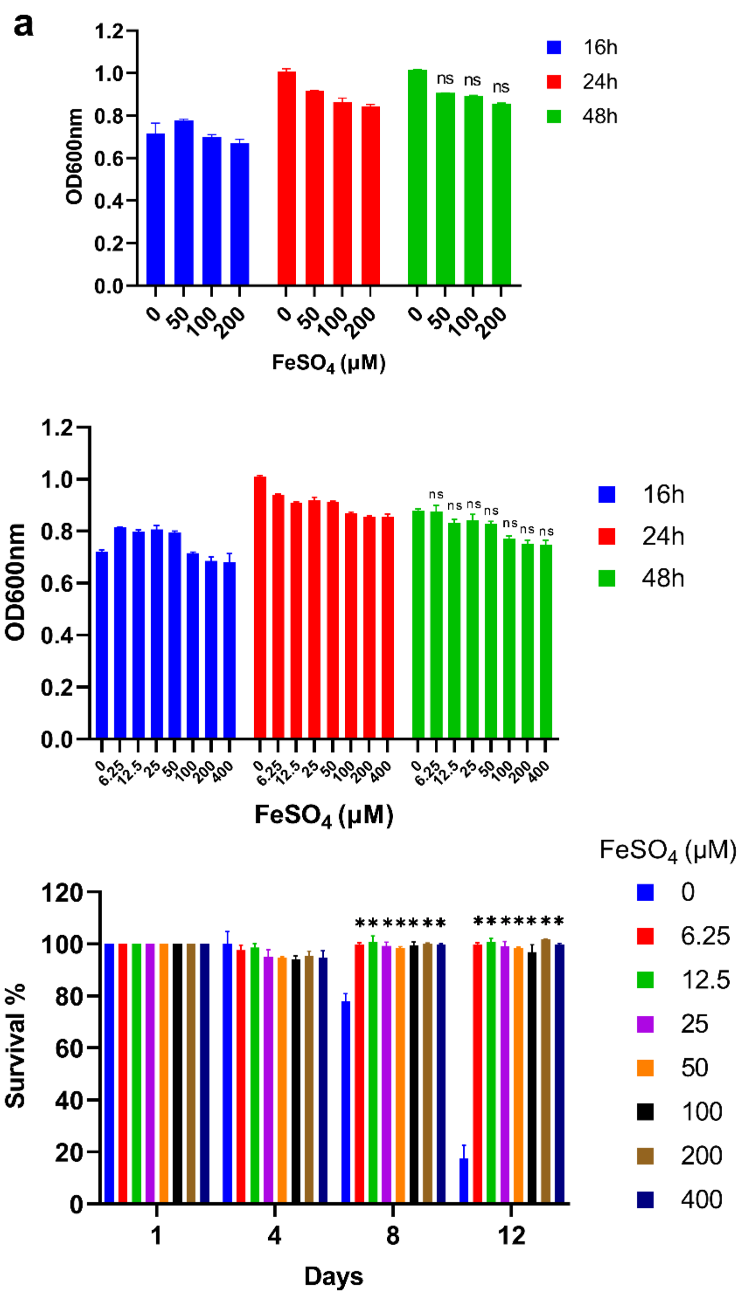


**Figure S1. Growth and chronological lifespan analysis of iron supplemented yeast cells.** (a) Cell growth analysis of experiments discussed in fig. 1. Cell growth OD600nm was measured at time points 16 h, 24 h, and 48 h using a microplate reader. (b) Growth of cells incubated with different concentrations of $FeSO_4$. (c) CLS of cells supplemented with different concentrations of $FeSO_4$. Statistical significance (*$p < 0.05$) was determined by Student's t-test.

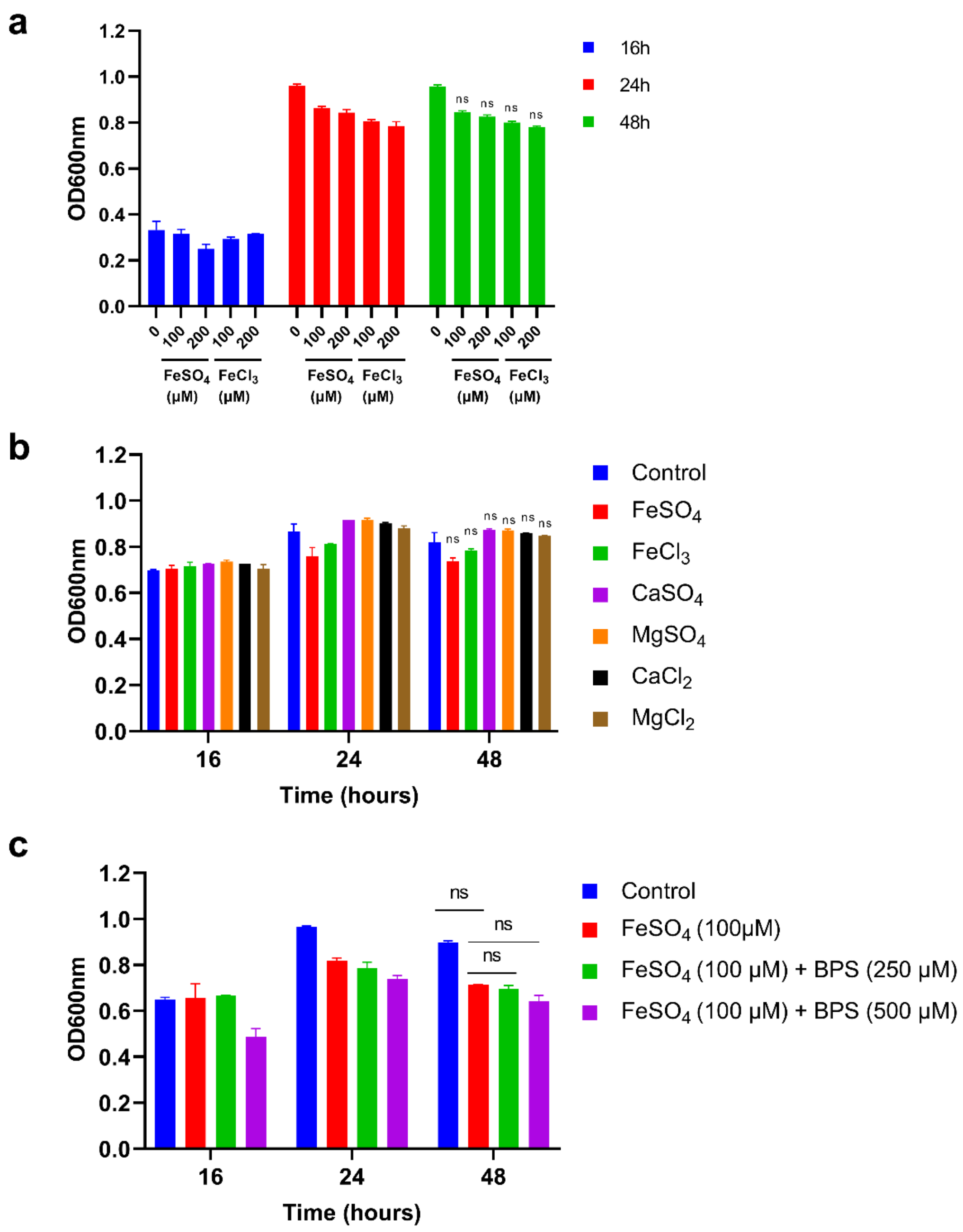


**Figure S2. Growth analysis of different iron, sulfate and chloride-containing salts supplemented yeast cells.** Cell growth analysis of experiments discussed in fig. 1b, 1c and 1d. Cell growth OD600nm was measured at time points 16 h, 24 h, and 48 h using a microplate reader. (a) Growth of cells incubated with different concentrations of $FeSO_4$ and $FeCl_3$. (b) Growth of cells incubated with 100 μM of $FeSO_4$, $FeCl_3$, $CaSO_4$, $MgSO_4$, $CaCl_2$, and $MgCl_2$. (c) Growth of cells incubated with 100 μM of $FeSO_4$ in the presence of different concentrations of BPS. Statistical significance (*$p < 0.05$) was determined by Student's t-test.

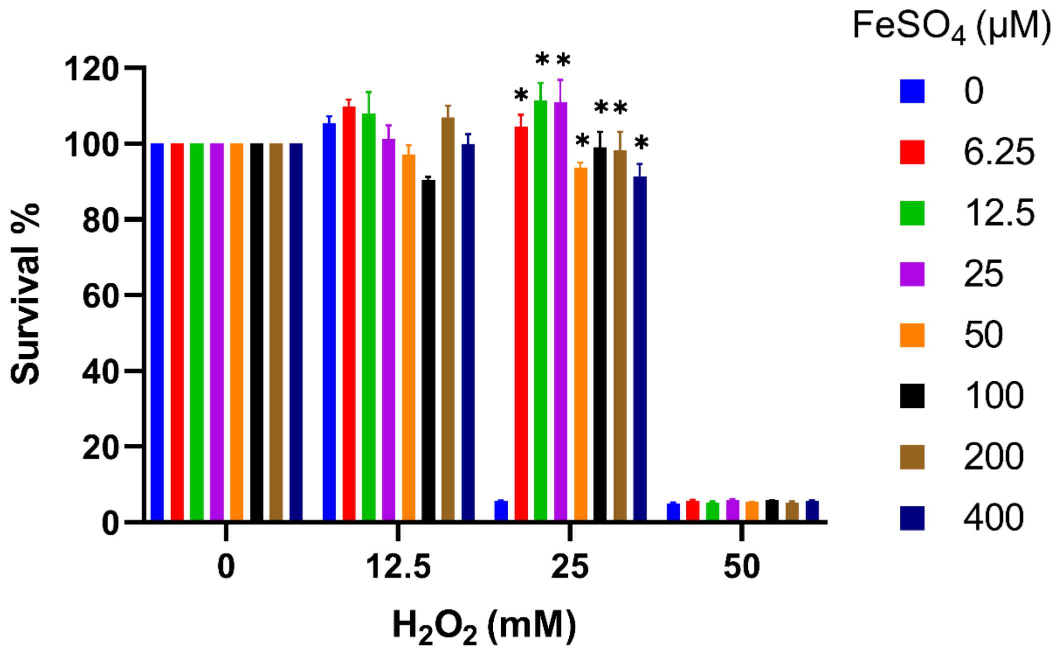


**Figure S3. Oxidative stress resistance analysis of iron supplemented yeast cells.** The prototrophic yeast strain was incubated with different concentrations of $FeSO_4$ and grown to stationary phase stage in SD medium for 72 h at 30 °C. After that, cells were diluted to OD600nm ~0.2 in YPD medium with different concentrations of $H_2O_2$ and grew at 30 °C. Oxidative stress phenotype was analyzed by comparing the cell growth of $H_2O_2$ treated cells with non-treated control. Statistical significance (*$p < 0.05$) was determined by Student's t-test.

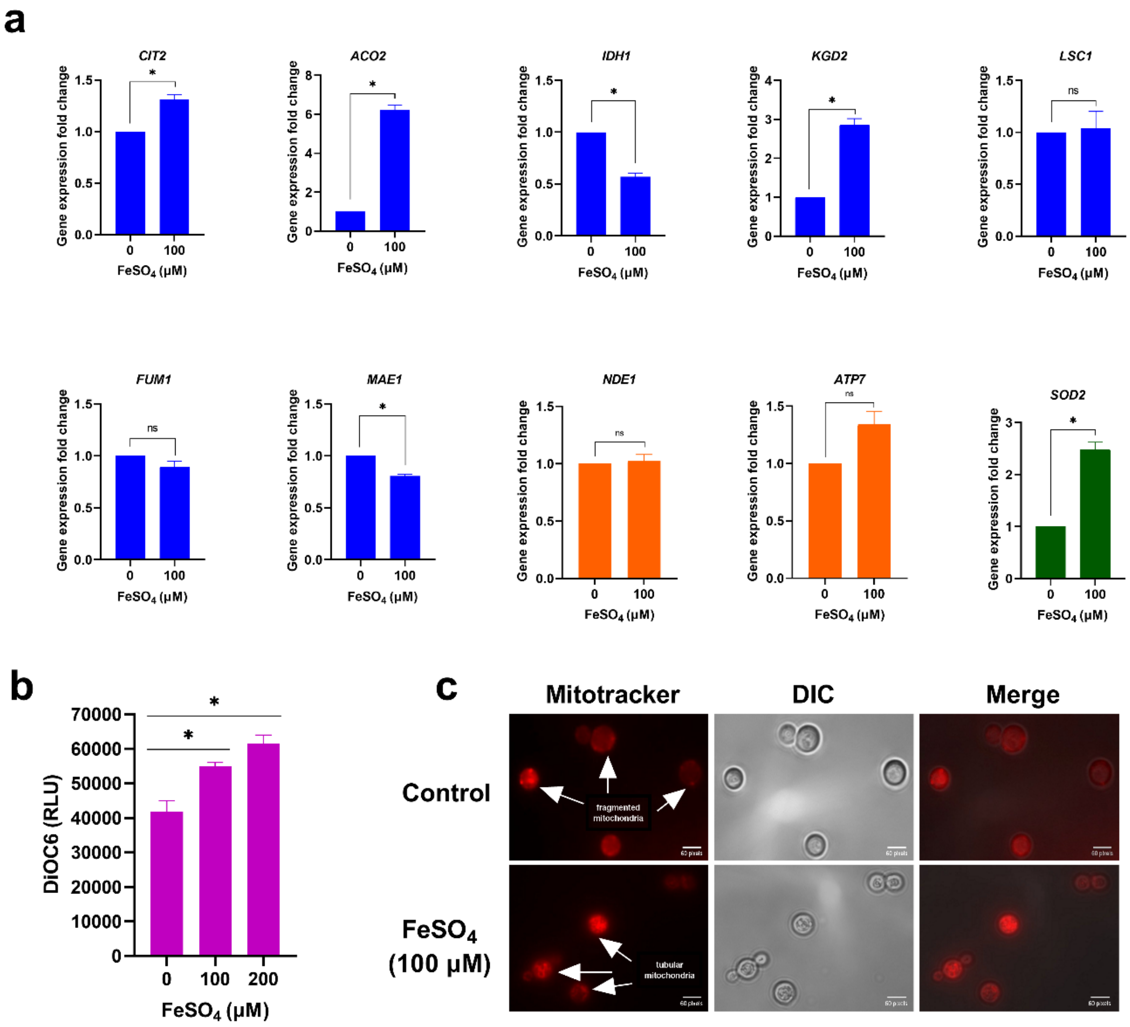


**Figure S4. Mitochondrial gene expression, membrane potential and structure analysis of iron supplemented yeast cells.** (a) Gene expression analysis of experiments discussed in fig. 3. Quantitative RT-PCR analysis of TCA genes, ETC genes (*NDE1* and *ATP7*), and *SOD2* gene. (b) Determination of mitochondrial membrane potential of cells incubated with indicated concentrations of $FeSO_4$. The yeast strain was incubated with different concentrations of $FeSO_4$ and grown to stationary phase stage in SD medium for 72 h at 30 $^oC$. Samples were stained with DiOC6(3) and fluorescence reading (excitation at 482nm, emission at 504nm) were measured by the microplate reader. (c) Mitochondrial structure analysis of control and iron supplemented cells. Cells were stained with MitoTracker™ Deep Red and samples were visualized with fluorescence microscope at 100x magnification. The fluorescence intensity of each sample was normalized with OD600nm. Statistical significance (*$p < 0.05$) was determined by Student's t-test.

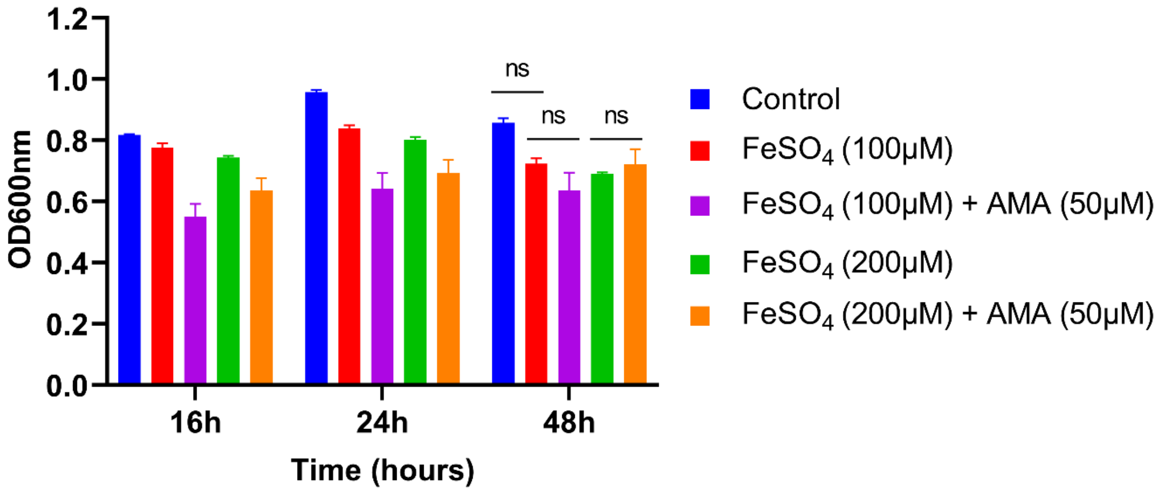


**Figure S5. Growth analysis of iron and antimycin A supplemented yeast cells.** Cell growth analysis of experiments discussed in fig. 4. Growth of cells (OD600nm) incubated with varying concentrations of $FeSO_4$ and 50 μM antimycin A (AMA) was measured at time points 16 h, 24 h, and 48 h using a microplate reader. Statistical significance (*$p < 0.05$) was determined by Student's t-test.
